# Asymmetrically interacting spreading dynamics on complex layered networks

**DOI:** 10.1038/srep05097

**Published:** 2014-05-29

**Authors:** Wei Wang, Ming Tang, Hui Yang, Younghae Do, Ying-Cheng Lai, GyuWon Lee

**Affiliations:** 1 Web Sciences Center, University of Electronic Science and Technology of China, Chengdu 610054, China; 2 Center for Atmospheric Remote Sensing(CARE), Kyungpook National University, Daegu, 702-701, South Korea; 3 Department of Mathematics, Kyungpook National University, Daegu 702-701, South Korea; 4 School of Electrical, Computer and Energy Engineering, Arizona State University, Tempe, Arizona 85287, USA; 5 Department of Astronomy and Atmospheric Sciences, Center for Atmospheric Remote Sensing(CARE), Kyungpook National University, Daegu, 702-701, South Korea

## Abstract

The spread of disease through a physical-contact network and the spread of information about the disease on a communication network are two intimately related dynamical processes. We investigate the asymmetrical interplay between the two types of spreading dynamics, each occurring on its own layer, by focusing on the two fundamental quantities underlying any spreading process: epidemic threshold and the final infection ratio. We find that an epidemic outbreak on the contact layer can induce an outbreak on the communication layer, and information spreading can effectively raise the epidemic threshold. When structural correlation exists between the two layers, the information threshold remains unchanged but the epidemic threshold can be enhanced, making the contact layer more resilient to epidemic outbreak. We develop a physical theory to understand the intricate interplay between the two types of spreading dynamics.

Epidemic spreading^1^,^2^,^3^,^4^,^5^,^6^ and information diffusion^7^,^8^,^9^,^10^ are two fundamental types of dynamical processes on complex networks. While traditionally these processes have been studied independently, in real-world situations there is always coupling or interaction between them. For example, whether large-scale outbreak of a disease can actually occur depends on the spread of information about the disease. In particular, when the disease begins to spread initially, individuals can become aware of the occurrence of the disease in their neighborhoods and consequently take preventive measures to protect themselves. As a result, the extent of the disease spreading can be significantly reduced^11^,^12^,^13^. A recent example is the wide spread of severe acute respiratory syndrome (SARS) in China in 2003, where many people took simple but effective preventive measures (e.g., by wearing face masks or staying at home) after becoming aware of the disease, even before it has reached their neighborhoods^14^. To understand how information spreading can mitigate epidemic outbreaks, and more broadly, the interplay between the two types of spreading dynamics has led to a new direction of research in complex network science^15^.

A pioneering step in this direction was taken by Funk *et al.*, who presented an epidemiological model that takes into account the spread of awareness about the disease^16^,^17^. Due to information diffusion, in a well-mixed population, the size of the epidemic outbreak can be reduced markedly. However, the epidemic threshold can be enhanced only when the awareness is sufficiently strong so as to modify the key parameters associated with the spreading dynamics such as the infection and recovery rates. A reasonable setting to investigate the complicated interplay between epidemic spreading and information diffusion is to assume two interacting network layers of of identical set of nodes, one for each type of spreading dynamics. Due to the difference in the epidemic and information spreading processes, the connection patterns in the two layers can in general be quite distinct. For the special case where the two-layer overlay networks are highly correlated in the sense that they have completely overlapping links and high clustering coefficient, a locally spreading awareness triggered by the disease spreading can raise the threshold even when the parameters in the epidemic spreading dynamics remain unchanged^16^,^17^. The situation where the two processes spread successively on overlay networks was studied with the finding that the outbreak of information diffusion can constrain the epidemic spreading process^18^. An analytical approach was developed to provide insights into the symmetric interplay between the two types of spreading dynamics on layered networks^19^. A model of competing epidemic spreading over completely overlapping networks was also proposed and investigated, revealing a coexistence regime in which both types of spreading can infect a substantial fraction of the network^20^.

While the effect of information diffusion (or awareness) on epidemic spreading has attracted much recent interest^21^,^22^,^23^,^24^,^25^,^26^,^27^,^28^, many outstanding issues remain. In this paper we address the following three issues. The first concerns the network structures that support the two types of spreading dynamics, which were assumed to be identical in some existing works. However, in reality, the two networks can differ significantly in their structures. For example, in a modern society, information is often transmitted through electronic communication networks such as telephones^29^ and the Internet^30^, but disease spreading usually takes place on a physical contact network^31^. The whole complex system should then be modeled as a double-layer coupled network (overlay network or multiplex network)^32^,^33^,^34^,^35^,^36^, where each layer has a distinct internal structure and the interplay between between the two layers has diverse characteristics, such as inter-similarity^37^, multiple support dependence^38^, and inter degree-degree correlation^39^, etc. The second issue is that the effects of one type of spreading dynamics on another are typically asymmetric^21^, requiring a modification of the symmetric assumption used in a recent work^19^. For example, the spread of a disease can result in elevated crisis awareness and thus facilitate the spread of the information about the disease^17^, but the spread of the information promotes more people to take preventive measures and consequently suppresses the epidemic spreading^26^. The third issue concerns the timing of the two types of spreading dynamics because they usually occur simultaneously on their respective layers and affect each other dynamically during the same time period^19^.

Existing works treating the above three issues separately showed that each can have some significant effect on the epidemic and information spreading dynamics^16^,^19^,^40^. However, a unified framework encompassing the sophisticated consequences of all three issues is lacking. The purpose of this paper is to develop an asymmetrically interacting spreading-dynamics model to integrate the three issues so as to gain deep understanding into the intricate interplay between the epidemic and information spreading dynamics. When all three issues are taken into account simultaneously, we find that an epidemic outbreak on the contact layer can induce an outbreak on the communication layer, and information spreading can effectively raise the epidemic threshold, making the contact layer more resistant to disease spreading. When inter-layer correlation exists, the information threshold remains unchanged but the epidemic threshold can be enhanced, making the contact layer more resilient to epidemic outbreak. These results are established through analytic theory with extensive numerical support.

## Results

In order to present our main results, we describe our two-layer network model and the dynamical process on each layer. We first treat the case where the double-layer networks are uncorrelated. We then incorporate layer-to-layer correlation in our analysis.

### Model of communication-contact double-layer network

Communication-contact coupled layered networks are one class of multiplex networks^41^. In such a network, an individual (a node) not only connects with his/her friends on a physical contact layer (subnetwork), but also communicates with them through the (electronic) communication layer. The structures of the two layers can in general be quite different. For example, an indoor-type of individual has few friends in the real world but may have many friends in the cyber space, leading to a much higher degree in the communication layer than in the physical-contact layer. Generally, the degree-to-degree correlation between the two layers cannot be assumed to be strong.

Our correlated network model of communication-contact layers is constructed, as follows. Two subnetworks *A* and *B* with the same node set are first generated independently, where *A* and *B* denote the communication and contact layers, respectively. Each layer possesses a distinct internal structure, as characterized by measures such as the mean degree and degree distribution. Then each node of layer *A* is matched one-to-one with that of layer *B* according to certain rules.

In an uncorrelated double-layer network, the degree distribution of one layer is completely independent of the distributions of other layer. For example, a hub node with a large number of neighbors in one layer is not necessarily a hub node in the other layer. In contrast, in a correlated double-layer network, the degree distributions of the two layers are strongly dependent upon each other. In a perfectly correlated double-layer network, hub nodes in one layer must simultaneously be hub nodes in the other layer. Quantitatively, the Spearman rank correlation coefficient^39^,^42^ *m_s_*, where *m_s_* ∈ [−1, 1] (see definition in **Methods**), can be used to characterize the degree correlation between the two layers. For *m_s_* > 0, the greater the correlation coefficient, the larger degree a pair of counterpart nodes can have. For *m_s_* < 0, as |*m_s_*| is decreased, a node of larger degree in one layer is matched with a node of smaller degree in the other layer.

### Asymmetrically interacting spreading dynamics

The dynamical processes of disease and information spreading are typically asymmetrically coupled with each other. The dynamics component in our model can be described, as follows. In the communication layer (layer *A*), the classic susceptible-infected-recovered (SIR) epidemiological model^43^ is used to describe the dissemination of information about the disease. In the SIR model, each node can be in one of the three states: (1) susceptible state (*S*) in which the individual has not received any information about the disease, (2) informed state(*I*), where the individual is aware of disease and is capable of transmitting the information to other individuals in the same layer, and (3) refractory state (*R*), in which the individual has received the information but is not willing to pass it on to other nodes. At each time step, the information can propagate from every informed node to all its neighboring nodes. If a neighbor is in the susceptible state, it will be informed with probability *β_A_*. At the same time, each informed node can enter the recovering phase with probability *µ_A_*. Once an informed node is recovered, it will remain in this state for all subsequent time. A node in layer *A* will get the information about the disease once its counterpart node in layer *B* is infected. As a result, dissemination of the information over layer *A* is facilitated by disease transmission on layer *B*.

**Figure 1:**
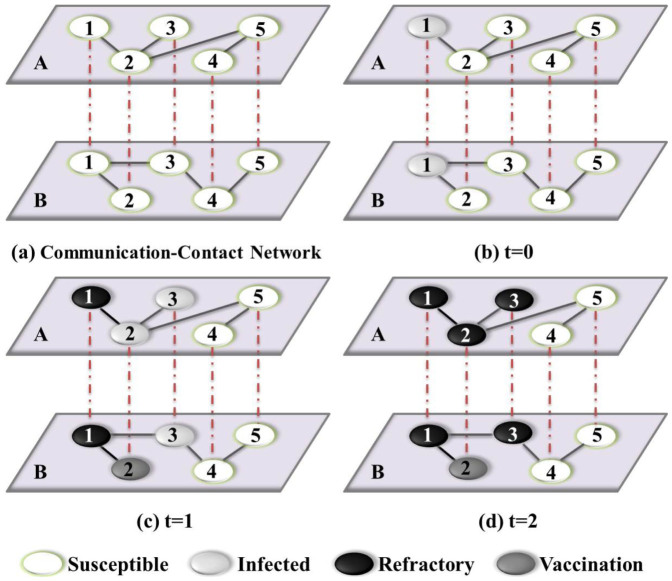
Illustration of asymmetrically coupled spreading processes on a simulated communication-contact double-layer network. (a) Communication and contact networks, denoted as layer *A* and layer *B*, respectively, each of five nodes. (b) At *t* = 0, node *B*_1_ in layer *B* is randomly selected as the initial infected node and its counterpart, node *A*_1_ in layer *A*, gains the information that *B*_1_ is infected, while all other pairs of nodes, one from layer *A* and another from layer *B*, are in the susceptible state. (c) At *t* = 1, within layer *A* the information is transmitted from *A*_1_ to *A*_2_ with probability *β_A_*. Node *B*_3_ in layer *B* can be infected by node *B*_1_ with probability *β_B_* and, if it is indeed infected, its corresponding node *A*_3_ in layer *A* gets the information as well. Since, by this time, *A*_2_ is already aware of the infection spreading, its counterpart *B*_2_ in layer *B* is vaccinated, say with probability *p*. At the same time, node *A*_1_ in layer *A* and its counterpart *B*_1_ in layer *B* enter into the refractory state with probability *µ_A_* and *µ_B_*, respectively. (d) At *t* = 2, all infected (or informed) nodes in both layers can no longer infect others, and start recovering from the infection. In both layers, the spreading dynamics terminate by this time.

The spreading dynamics in layer *B* can be described by the SIRV model^26^, in which a fourth sate, the state of vaccination (*V*), is introduced. Mathematically, the SIR component of the spreading dynamics is identical to the dynamics on layer *A* except for different infection and recovery rates, denoted by *β_B_* and *µ_B_*, respectively. If a node in layer *B* is in the susceptible state but its counterpart node in layer *A* is in the infected state, the node in layer *B* will be vaccinated with probability *p*. Disease transmission in the contact layer can thus be suppressed by dissemination of information in the communication layer. The two spreading processes and their dynamical interplay are illustrated schematically in Fig. 1. Without loss of generality, we set *µ_A_* = *µ_B_* = 1.

### Theory of spreading dynamics in uncorrelated double-layer networks

Two key quantities in the dynamics of spreading are the outbreak threshold and the fraction of infected nodes in the final steady state. We develop a theory to predict these quantities for both information and epidemic spreading in the double-layer network. In particular, we adopt the heterogeneous mean-field theory^44^ to uncorrelated double-layer networks.

Let *P_A_*(*k_A_*) and *P_B_*(*k_B_*) be the degree distributions of layers *A* and *B*, with mean degree 〈*k_A_*〉 and 〈*k_B_*〉, respectively. We assume that the subnetworks associated with both layers are random with no degree correlation. The time evolution of the epidemic spreading is described by the variables 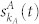, 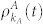, and 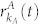, which are the densities of the susceptible, informed, and recovered nodes of degree *k_A_* in layer *A* at time *t*, respectively. Similarly, 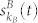, 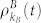, 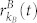, and 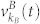 respectively denote the susceptible, infected, recovered, and vaccinated densities of nodes of degree *k_B_* in layer *B* at time *t*.

The mean-field rate equations of the information spreading in layer *A* are 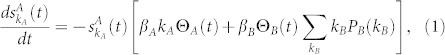

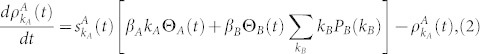

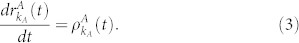
The mean-field rate equations of epidemic spreading in layer *B* are given by 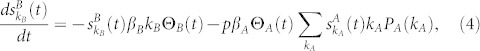

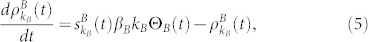


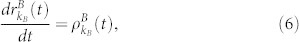

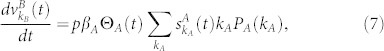
where Θ*_A_*(*t*) (Θ*_B_*(*t*)) is the probability that a neighboring node in layer A (layer B) is in the informed (infected) state (See **Methods** for details).

From Eqs. (1)–(7), the density associated with each distinct state in layer *A* or *B* is given by 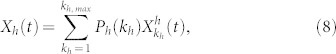
where *h* ∈ {*A*, *B*}, *X* ∈ {*S*, *I*, *R*, *V*}, and *k_h_*_,*max*_ denotes the largest degree of layer *h*. The final densities of the whole system can be obtained by taking the limit *t* → ∞.

Due to the complicated interaction between the disease and information spreading processes, it is not feasible to derive the exact threshold values. We resort to a linear approximation method to get the outbreak threshold of information spreading in layer *A* (see Supporting Information for details) as 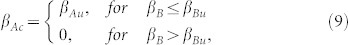
where 
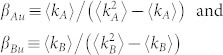
denote the outbreak threshold of information spreading in layer *A* when it is isolated from layer *B*, and that of epidemic spreading in layer *B* when the coupling between the two layers is absent, respectively.

Equation (9) has embedded within it two distinct physical mechanisms for information outbreak. The first is the intrinsic information spreading process on the isolated layer *A* without the impact of the spreading dynamics from layer *B*. For *β_B_* > *β_Bu_*, the outbreak of epidemic will make a large number of nodes in layer *A* “infected” with the information, even if on layer *A*, the information itself cannot spread through the population efficiently. In this case, the information outbreak has little effect on the epidemic spreading in layer *B* because very few nodes in this layer are vaccinated. We thus have *β_Bc_* ≈ *β_Bu_* for *β_A_* ≤ *β_Au_*.

However, for *β_A_* > *β_Au_*, epidemic spreading in layer *B* is restrained by information spread, as the informed nodes in layer *A* tend to make their counterpart nodes in layer *B* vaccinated. Once a node is in the vaccination state, it will no longer be infected. In a general sense, vaccination can be regarded as a type of “disease,” as every node in layer *B* can be in one of the two states: infected or vaccinated. Epidemic spreading and vaccination can thus be viewed as a pair of competing “diseases” spreading in layer *B*^20^. As pointed out by Karrer and Newman^20^, in the limit of large network size *N*, the two competing diseases can be treated as if they were in fact spreading non-concurrently, one after the other.

Initially, both epidemic and vaccination spreading processes exhibit exponential growth (see Supporting Information). We can thus obtain the ratio of their growth rates as 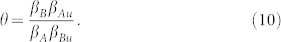
For *θ* > 1, the epidemic disease spreads faster than the vaccination. In this case, the vaccination spread is insignificant and can be neglected. For *θ* < 1, information spreads much faster than the disease, in accordance with the situation in a modern society. Given that the vaccination and epidemic processes can be treated successively and separately, the epidemic outbreak threshold can be derived by a bond percolation analysis^20^,^45^ (see details in Supporting Information). We obtain 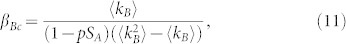
where *S_A_* is the density of the informed population, which can be obtained by solving Eqs. (S18) and (S19) in Supporting Information. For *θ* < 1, we see from Eq. (11) that the threshold for epidemic outbreak can be enhanced by the following factors: strong heterogeneity in the communication layer, large information-transmission rate, and large vaccination rate.

### Simulation results for uncorrelated networks

We use the standard configuration model to generate networks with power-law degree distributions^46^,^47^,^48^ for the communication subnetwork (layer A). The contact subnetwork in layer *B* is of the Erdős and Rényi (ER) type^49^. We use the notation SF-ER to denote the double-layer network. The sizes of both layers are set to be *N_A_* = *N_B_* = 2 × 10^4^ and their average degrees are 〈*k_A_*〉 = 〈*k_B_*〉 = 8. The degree distribution of the communication layer is 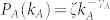 with the coefficient 
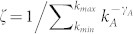
 and the maximum degree 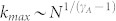. We focus on the case of *γ_A_* = 3.0 here in the main text (the results for other values of the exponent, e.g., *γ_A_* = 2.7 and 3.5, are similar, which are presented in Supporting Information). The degree distribution of the contact layer is 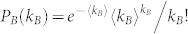. To initiate an epidemic spreading process, a node in layer *B* is randomly infected and its counterpart node in layer *A* is thus in the informed state, too. We implement the updating process with parallel dynamics, which is widely used in statistical physics^50^ (see Sec. S3A in Supporting Information for more details). The spreading dynamics terminates when all infected nodes in both layers are recovered, and the final densities *R_A_*, *R_B_*, and *V_B_* are then recorded.

**Figure 2:**
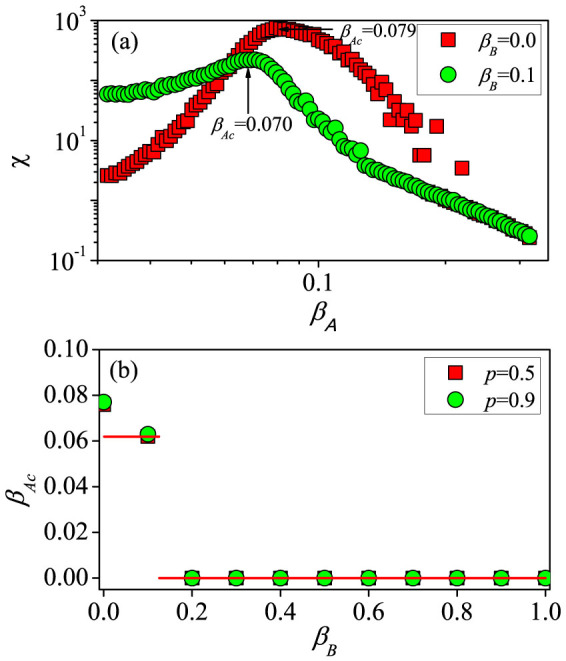
The identification of epidemic threshold on SF-ER networks. (a) The susceptibility measure *χ* as a function of the information-transmission rate *β_A_* for *p* = 0.5, *β_B_* = 0.0 (red squares) and *β_B_* = 0.1 (green circles), (b) the threshold *β_Ac_* of information spreading as a function of the disease-transmission rate *β_B_* for vaccination rate *p* = 0.5 (red squares) and *p* = 0.9 (green circles), where the red solid lines are analytical predictions from Eq. (9).

For epidemiological models [e.g., the susceptible-infected-susceptible (SIS) and SIR] on networks with a power-law degree distribution, the finite-size scaling method may not be effective to determine the critical point of epidemic dynamics^51^,^52^, because the outbreak threshold depends on network size and it goes to zero in the thermodynamic limit^43^,^53^. Therefore, we employ the *susceptibility measure*^52^ *χ* to numerically determine the size-dependent outbreak threshold: 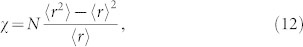
where *N* is network size (*N* = *N_A_* = *N_B_*), and *r* denotes the final outbreak ratio such as the final densities *R_A_* and *R_B_* of the recovered nodes in layers *A* and *B*, respectively. We use 2 × 10^3^ independent dynamic realizations on a fixed double-layer network to calculate the average value of *χ* for the communication layer for each value of *β_A_*. As shown in Fig. 2(a), *χ* exhibits a maximum value at *β_Ac_*, which is the threshold value of the information spreading process. The simulations are further implemented using 30 different two-layer network realizations to obtain the average value of *β_Ac_*. The identical simulation setting is used for all subsequent numerical results, unless otherwise specified. Figure 2(b) shows the information threshold *β_Ac_* as a function of the disease-transmission rate *β_B_*. Note that the statistical errors are not visible here (same for similar figures in the paper), as they are typically vanishingly small. We see that the behavior of the information threshold can be classified into two classes, as predicted by Eq. (9). In particular, for *β_B_* ≤ *β_Bu_* = 1/〈*k_B_*〉 = 0.125, the disease transmission on layer *B* has little impact on the information threshold on layer *A*, as we have 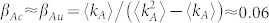. For *β_B_* > *β_Bu_*, the outbreak of epidemic on layer *B* leads to *β_Ac_* = 0.0. Comparison of the information thresholds for different vaccination rates shows that the value of the vaccination probability *p* has essentially no effect on *β_Ac_*.

**Figure 3:**
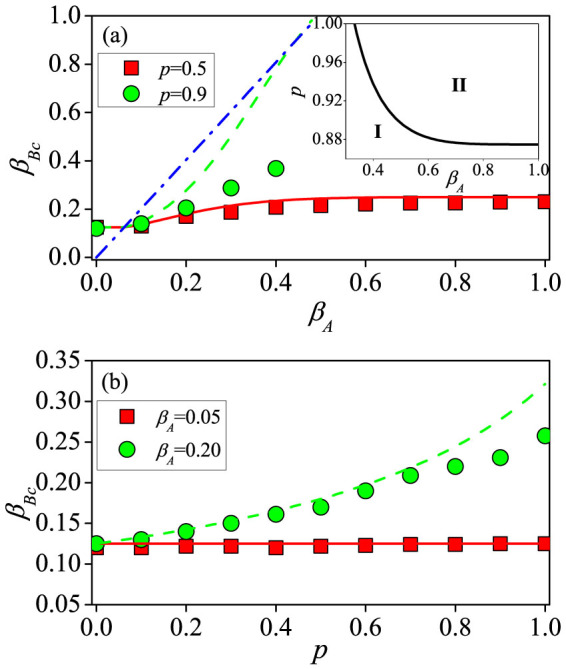
For SF-ER double-layer networks, epidemic threshold *β_Bc_* as a function of the information-transmission rate *β_A_* (a) and the vaccination rate *p* (b). In (a), the red solid (*p* = 0.5) and green dashed (*p* = 0.9) lines are the analytical predictions from Eq. (11), and the blue dot-dashed line denotes the case of *θ* = 1 from Eq. (10). The inset of (a) shows the condition under which a giant residual cluster of layer *B* exists [from Eq. (S20) in Supporting Information] in phase I. In (b), the red solid line (*β_A_* = 0.05) corresponds to *β_Bc_* = *β_Bu_*, and the green dashed line (*β_A_* = 0.20) is the analytical prediction from Eq. (11).

Figure 3 shows the effect of the information-transmission rate *β_A_* and the vaccination rate *p* on the epidemic threshold *β_Bc_*. From Fig. 3(a), we see that the value of *β_Bc_* is not influenced by *β_A_* for *β_A_* ≤ *β_Au_* ≈ 0.06, whereas *β_Bc_* increases with *β_A_*. For *p* = 0.5, the analytical results from Eq. (11) are consistent with the simulated results. However, deviations occur for larger values of *p*, e.g., *p* = 0.9, because the effect of information spreading is over-emphasized in cases where the two types of spreading dynamics are treated successively but not simultaneously. The gap between the theoretical and simulated thresholds diminishes as the network size is increased, validating applicability of the analysis method that, strictly speaking, holds only in the thermodynamic limit^20^ (see details in Supporting Information). Note that a giant residual cluster does not exist in layer *B* for *p* = 0.9 and *β_A_* ≥ 0.49, ruling out epidemic outbreak. The phase diagram indicating the possible existence of a giant residual cluster [Eq. (S20) in Supporting Information] is shown in the inset of Fig. 3(a), where in phase II, there is no such cluster. In Fig. 3(b), a large value of *p* causes *β_Bc_* to increase for *β_A_* > *β_Au_*. We observe that, similar to Fig. 3(a), for relatively large values of *p*, say *p* ≥ 0.8, the analytical prediction deviates from the numerical results. The effects of network size *N*, exponent *γ_A_* and SF-SF network structure on the information and epidemic thresholds are discussed in detail in Supporting Information.

**Figure 4:**
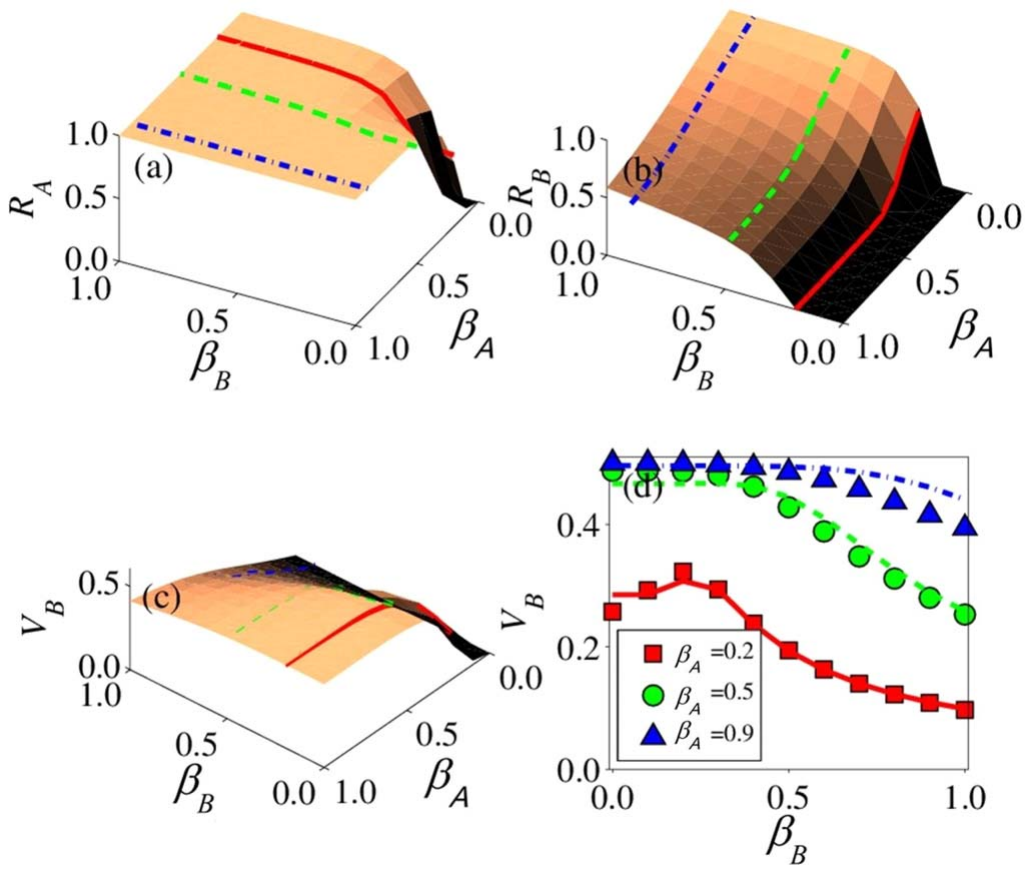
For SF-ER networks, the final density in each state versus the parameters *β_A_* and *β_B_*: (a) recovered density *R_A_*, (b) recovered density *R_B_*, (c) the vaccination density *V_B_*, and (d) *V_B_* versus *β_B_* for *β_A_* = 0.2, 0.5, 0.9. The value of parameter *p* is 0.5. Different lines are the numerical solutions of Eqs. (1) – (8) in the limit *t* → ∞. In (a) and (d), we select three different values of *β_A_* (0.2, 0.5, and 0.9), corresponding to the red solid, green dashed, and blue dot-dashed lines, respectively. In (b) and (c), three different values of *β_B_* are chosen (0.2, 0.5, and 0.9), corresponding to the red solid, green dashed, and blue dot-dashed lines, respectively.

The final dynamical state of the double-layer spreading system is shown in Fig. 4. From Fig. 4(a), we see that the final recovered density *R_A_* for information increases with *β_A_* and *β_B_* rapidly for *β_A_* ≤ *β_Au_* and *β_B_* ≤ *β_Bu_*. Figure 4(b) reveals that the recovered density *R_B_* for disease decreases with *β_A_*. We see that a large value of *β_A_* can prevent the outbreak of epidemic for small values of *β_B_*, as *R_B_* → 0 for *β_B_* = 0.2 and *β_A_* ≥ 0.5 (the red solid line). From Fig. 4(c), we see that, with the increase in *β_A_*, more nodes in layer *B* are vaccinated. It is interesting to note that the vaccinated density *V_B_* exhibits a maximum value if *β_A_* is not large. Figure 4 shows that the maximum value of *V_B_* is about 0.32, which occurs at *β_B_* ≈ 0.20, for *β_A_* = 0.2. Combining with Fig. 3(a), we find that the corresponding point of the maximum value *β_B_* ≈ 0.20 is close to *β_Bc_* ≈ 0.16 for *p* = 0.5. This is because the transmission of disease has the opposite effects on the vaccinations. For *β_B_* ≤ *β_Bc_*, the newly infected nodes in layer *B* will facilitate information spreading in layer *A*, resulting in more vaccinated nodes. For *β_B_* > *β_Bc_*, the epidemic spreading will make a large number of nodes infected, reducing the number of nodes that are potentially to be vaccinated. For relatively large values of *β_A_*, information tends to spread much faster than the disease for *β_B_* ≤ *β_Bc_*, e.g., *θ* ≈ 0.21 for *β_A_* = 0.5, *p* = 0.5, *β_Bc_* ≈ 0.22, and *θ* ≈ 0.12 for *β_A_* = 0.9, *p* = 0.5, and *β_Bc_* ≈ 0.23. In this case, the effect of disease transmission on information spreading is negligible. The densities of the final dynamical states for SF-SF networks are also shown in Supporting Information, and we observe similar behaviors.

### Spreading dynamics on correlated double-layer networks

**Figure 5:**
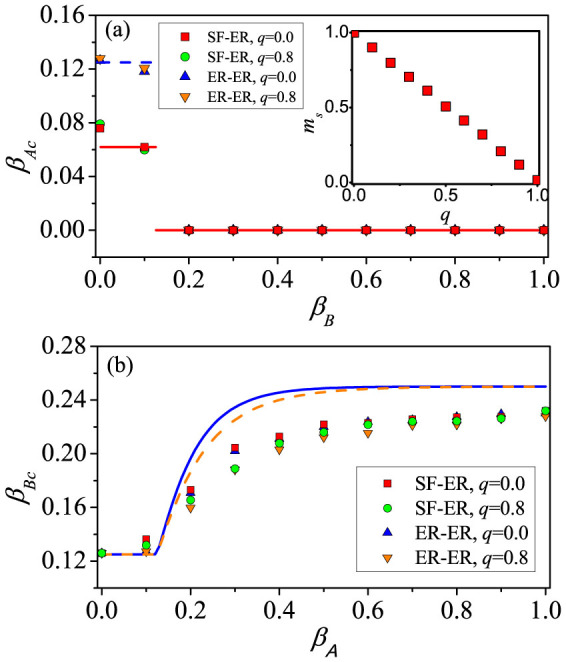
For two-layer correlated networks with vaccination probability *p* = 0.5, the effect of one type of spreading dynamics on the outbreak threshold of the counter-type spreading dynamics. (a) *β_Ac_* versus *β_B_* on SF-ER networks with *q* = 0.0 (red squares) and *q* = 0.8 (green circles), and ER-ER networks with *q* = 0.0 (blue up triangles) and *q* = 0.8 (orange down triangles). Red solid (SF-ER) and blue dashed (ER-ER) lines are the analytical predictions from Eq. (9) and Eq. (S37) (in Supporting Information), respectively. The inset shows the inter-layer correlation *m_s_* as a function of rematching probability *q*. (b) *β_Bc_* versus *β_A_* on SF-ER networks with *q* = 0.0 (red squares) and *q* = 0.8 (green circles), and ER-ER networks with *q* = 0.0 (blue up triangles) and *q* = 0.8 (orange down triangles). Blue solid (*q* = 0.0) and orange dashed (*q* = 0.8) lines are the analytical predictions for ER-ER networks from Eqs. (S38) – (S41) in Supporting Information.

In realistic multiplex networks certain degree of inter-layer correlations is expected to exist^35^. For example, in social networks, positive inter-layer correlation is more common than negative correlation^54^,^55^. That is, an “important” individual with a large number of links in one network layer (e.g., representing one type of social relations) tends to have many links in other types of network layers that reflect different kinds of social relations. Recent works have shown that inter-layer correlation can have a large impact on the percolation properties of multiplex networks^37^,^39^. Here, we investigate how the correlation between the communication and contact layers affects the information and disease spreading dynamics. To be concrete, we focus on the effects of positive correlation on the two types of spreading dynamics. It is necessary to construct a two-layer correlated network with adjustable degree of inter-layer correlation. This can be accomplished by first generating a two-layer network with the maximal positive correlation, where each layer has the same structure as uncorrelated networks. Then, *Nq* pairs of counterpart nodes, in which *q* is the rematching probability, are rematched randomly, leading to a two-layer network with weaker inter-layer correlation. The inter-layer correlation after rematching is given by (see **Methods**) 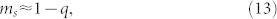
which is consistent with the numerical results [e.g., see inset of Fig. 5(a) below]. For SF-ER networks with fixed correlation coefficient, the mean-field rate equations of the double-layer system cannot be written down because the concrete expressions of the conditional probabilities *P*(*k_B_*|*k_A_*) and *P*(*k_A_*|*k_B_*) are no longer available.

**Figure 6:**
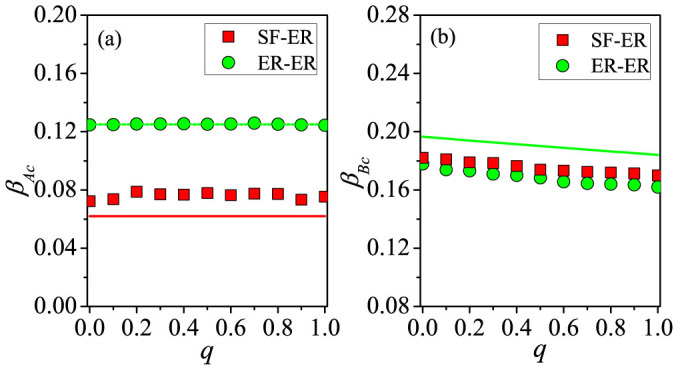
Effect of varying the rematching probability on outbreak thresholds of the two types of spreading dynamics. (a) *β_Ac_* versus *q* on SF-ER (red squares) and ER-ER networks (green circles) for *β_B_* = 0.05 and *p* = 0.5. Red Solid (SF-ER) and green dashed (ER-ER) lines are analytical predictions from Eq. (9) and Eq. (S37) in Supporting Information, respectively. (b) *β_Bc_* versus *q* on SF-ER (red squares) and ER-ER networks (green circles) for *β_A_* = 0.2 and *p* = 0.5. Green solid line is analytical prediction for ER-ER networks from Eqs. (S38) – (S41) in Supporting Information.

**Figure 7:**
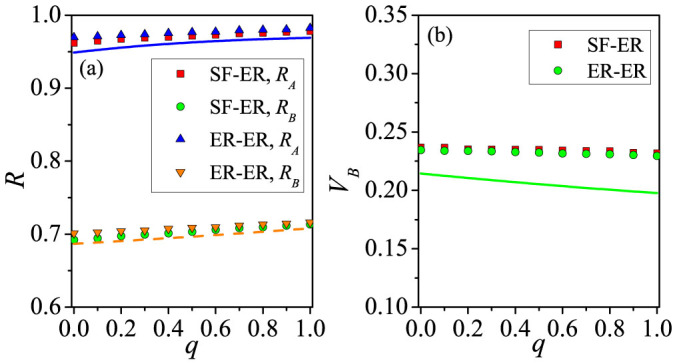
Effect of rematching probability on the final state. (a) *R_A_* versus *q* on SF-ER (red squares) and ER-ER networks (blue up triangles), *R_B_* versus *q* on SF-ER (green circles) and ER-ER networks (orange down triangles). (b) *V_B_* versus *q* on SF-ER (red squares) and ER-ER networks (green circles). Different lines represent the analytic solutions for ER-ER networks, calculated by summing the final densities of all degrees from Eqs. (S28) – (S34) in Supporting Information. The parameter setting is *β_A_* = 0.2, *β_B_* = 0.4 and *p* = 0.5.

We investigate how the rematching probability *q* affects the outbreak thresholds in both the communication and epidemic layers. As shown in Fig. 5, we compare the case of *q* = 0.8 with that of *q* = 0.0. From Fig. 5(a), we see that *q* has little impact on the outbreak threshold *β_Ac_* of the communication layer [with further support in Fig. 6(a), and analytic explanation using ER-ER correlated layered networks in Supporting Information]. We also see that the value of *β_Ac_* for ER-ER layered networks with the same mean degree is greater because of the homogeneity in the degree distribution of layer *A*. Figures 5(b) and 6(b) show that *β_Bc_* decreases with *q* or, equivalently, *β_Bc_* increases with *m_s_*. This is because stronger inter-layer correlation can increase the probability for nodes with large degrees in layer *B* to be vaccinated, thus effectively preventing the outbreak of epidemic [see also Eqs. (S38)–(S41) in Supporting Information]. Figure 7 shows the final densities of different populations, providing the consistent result that, with the increase (decrease) of *q* (*m_s_*), the final densities *R_A_* and *R_B_* increase but the density *V_B_* decreases. For SF-SF networks, we obtain similar results (shown in Supporting Information).

## Discussion

To summarize, we have proposed an asymmetrically interacting, double-layer network model to elucidate the interplay between information diffusion and epidemic spreading, where the former occurs on one layer (the communication layer) and the latter on the counterpart layer. A mean-field based analysis and extensive computations reveal an intricate interdependence of two basic quantities characterizing the spreading dynamics on both layers: the outbreak thresholds and the final fractions of infected nodes. In particular, on the communication layer, the outbreak of the information about the disease can be triggered not only by its own spreading dynamics but also by the the epidemic outbreak on the counter-layer. In addition, high disease and information-transmission rates can enhance markedly the final density of the informed or refractory population. On the layer of physical contact, the epidemic threshold can be increased but only if information itself spreads through the communication layer at a high rate. The information spreading can greatly reduce the final refractory density for the disease through vaccination. While a rapid spread of information will prompt more nodes in the contact layer to consider immunization, the authenticity of the information source must be verified before administrating large-scale vaccination.

We have also studied the effect of inter-layer correlation on the spreading dynamics, with the finding that stronger correlation has no apparent effect on the information threshold, but it can suppress the epidemic spreading through timely immunization of large-degree nodes^56^. These results indicate that it is possible to effectively mitigate epidemic spreading through information diffusion, e.g., by informing the high-centrality hubs about the disease.

The challenges of studying the intricate interplay between social and biological contagions in human populations are generating interesting science^57^. In this work, we study asymmetrically interacting information-disease dynamics theoretically and computationally, with implications to behavior-disease coupled systems and articulation of potential epidemic-control strategies. Our results would stimulate further works in the more realistic situation of asymmetric interactions.

During the final writing of this paper, we noted one preprint posted online studying the dynamical interplay between awareness and epidemic spreading in multiplex networks^58^. In that work, the two competing infectious strains are described by two SIS processes. The authors find that the epidemic threshold depends on the topological structure of the multiplex network and the interrelation with the awareness process by using a Markov-chain approach. Our work thus provides further understanding and insights into spreading dynamics on multi-layer coupled networks.

## Methods

### Mean-Field theory for the uncorrelated double-layer networks

To derive the mean-field rate equations for the density variables, we consider the probabilities that node *A_i_* in layer *A* and node *B_i_* in layer *B* become infected during the small time interval [*t*, *t* + *dt*]. On the communication layer, a susceptible node *A_i_* of degree *k_A_* can obtain the information in two ways: from its neighbors in the same layer and from its counterpart node in layer *B*. For the first route, the probability that node *A_i_* receives information from one of its neighbors is *k_A_β_A_*Θ*_A_*(*t*)*dt*, where Θ*_A_*(*t*) is the probability that a neighboring node is in the informed state^59^ and is given by 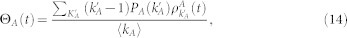
where 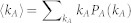. To model the second scenario, we note that, due to the asymmetric coupling between the two layers, a node in layer *A* being susceptible requires that its counterpart node in layer *B* be susceptible, too. A node in the communication layer will get the information about the disease once its counterpart node in layer *B* is infected, which occurs with the probability 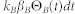, where *P*(*k_B_*|*k_A_*) denotes the conditional probability that a node of degree *k_A_* in layer *A* is linked to a node of degree *k_B_* in layer *B*, and *k_B_β*_B_Θ*_B_*(*t*)*dt* is the probability for a counterpart node of degree *k_B_* to become infected in the time interval [*t*, *t* + *dt*]. If the subnetworks in both layers are not correlated, we have *P*(*k_B_*|*k_A_*) = *P_B_*(*k_B_*). The mean-field rate equations of the information spreading in layer *A* are Eqs. (1)–(3).

On layer *B*, a susceptible node *B_i_* of degree *k_B_* may become infected or vaccinated in the time interval [*t*, *t* + *dt*]. This can occur in two ways. Firstly, it may be infected by a neighboring node in the same layer with the probability *k_B_β_B_*Θ*_B_*(*t*)*dt*, where Θ*_B_*(*t*) is the probability that a neighbor is in the infected state and is given by 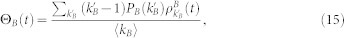
where 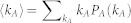. Secondly, if its counterpart node in layer *A* has already received the information from one of its neighbors, it will be vaccinated with probability *p*. The probability for a node in layer *B* to be vaccinated, taking into account the interaction between the two layers, is 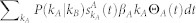, where *P*(*k_A_*|*k_B_*) denotes the conditional probability that a node of degree *k_B_* in layer *B* is linked to a node of degree *k_A_* in layer *A*, and 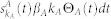 is the informed probability for the counterpart node of degree *k_A_* in the susceptible state [*P*(*k_A_*|*k_B_*) = *P_A_*(*k_A_*) for *m_s_* = 0]. The mean-field rate equations of epidemic spreading in layer *B* are Eqs. (4) – (7). We note that the second term on the right side of Eq. (4) does not contain the variable 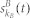 because a node in layer *B* must be in the susceptible state if its counterpart node in layer *A* is in the susceptible state.

### Spearman rank correlation coefficient

The correlation between the layers can be quantified by the Spearman rank correlation coefficient^39^,^42^ defined as 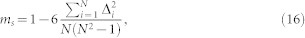
where *N* is network size and Δ*_i_* denotes the difference between node *i*'s degrees in the two layers. When a node in layer *A* is matched with a random node in layer *B*, *m_s_* is approximately zero in the thermodynamic limit. In this case, the double-layer network is uncorrelated^39^,^42^. When every node has the same rank of degree in both layers, we have *m_s_* ≈ 1. In this case, there is a maximally positive inter-layer correlation where, for example, the hub node with the highest degree in layer *A* is matched with the largest hub in layer *B*, and the same holds for the nodes with the smallest degree. In the case of maximally negative correlation, the largest hub in one layer is matched with a node having the minimal degree in the other layer, so we have *m_s_* ≈ −1.

In a double-layer network with the maximally positive correlation, any pair of nodes having the same rank of degree in the respective layers are matched, i.e., Δ*_i_* = 0 for any pair of nodes *A_i_* and *B_i_*. We thus have *m_s_* = 1, according to Eq. (16). After random rematching, a pair of nodes have Δ*_i_* = 0 with probability 1 − *q* and a random difference 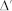 with probability *q*. Equation (16) can then be rewritten as 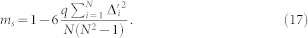
When all nodes are randomly rematched, the layers in the network are completely uncorrelated, i.e., *m_s_* ≈ 0. In this case, we have 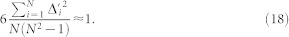
Submitting Eq. (18) into Eq. (17), the inter-layer correlation after rematching is given by 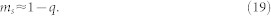


## Author Contributions

W.W., M.T. and Y.C.L. devised the research project. W.W. and H.Y. performed numerical simulations. W.W., M.T., Y.H.D. and Y.C.L. analyzed the results. W.W., M.T., Y.H.D., Y.C.L. and G.W.L. wrote the paper.

## Supplementary Material

Supplementary Information Supporting Information
