# Supplementary material for: Asymmetrically interacting spreading dynamics on complex layered networks
Source: Sci Rep. 2014 May 29;4:5097. doi: 10.1038/srep05097 (PMC4037715; doi:10.1038/srep05097)
Supplement: Supplementary Information — Supporting Information [file srep05097-s1.pdf]

Supporting Information for

Asymmetrically interacting spreading dynamics on complex layered networks

Wei Wang, Ming Tang, Hui Yang, Younghae Do, Ying-Cheng Lai and GyuWon Lee

Contents

S1. Spreading dynamics on uncorrelated doubule-layer networks 2
A. Mean-field rate equations 2
B. Linear analysis for the information threshold 2
C. Competing percolation theory for epidemic threshold 3

S2. Spreading dynamics on correlated double-layer networks 6
A. Mean-field rate equations 6
B. Linear analysis for the information threshold 6
C. Competing percolation theory for epidemic threshold 7

S3. Simulation results 8
A. Simulation process 8
B. Uncorrelated double-layer networks 8
C. Correlated double-layer networks 10

References 15

S1. Spreading dynamics on uncorrelated doubule-layer networks

We adopt the heterogeneous mean-field theory [1] to uncorrelated double-layer networks. Let $P_A(k_A)$ and $P_B(k_B)$ be the degree distributions of layers A and B, with mean degree $\langle k_A\rangle$ and $\langle k_B\rangle$, respectively. We assume that the subnetworks associated with both layers are random with no degree correlation. The time evolution of the epidemic spreading is described by the variables $s^A_{k_A}(t)$, $\rho^A_{k_A}(t)$, and $r^A_{k_A}(t)$, which are the densities of the susceptible, infected, and recovered nodes of degree k_A in layer A at time t, respectively. Similarly, $s^B_{k_B}(t)$, $\rho^B_{k_B}(t), r^B_{k_B}(t)$, and $v^B_{k_B}(t)$ respectively denote the susceptible, infected, recovered, and vaccinated densities of nodes of degree k_B in layer B at time t.

A. Mean-field rate equations

The mean-field rate equations of the information spreading in layer A are then

$$\frac{ds^A_{k_A}(t)}{dt} = -s^A_{k_A}(t)[\beta_A k_A \Theta_A(t) + \beta_B \Theta_B(t)\sum_{k_B} k_B P_B(k_B)], \tag{S1}$$

$$\frac{d\rho^A_{k_A}(t)}{dt} = s^A_{k_A}(t)[\beta_A k_A \Theta_A(t) + \beta_B \Theta_B(t)\sum_{k_B} k_B P_B(k_B)] - \rho^A_{k_A}(t), \tag{S2}$$

$$\frac{dr^A_{k_A}(t)}{dt} = \rho^A_{k_A}(t). \tag{S3}$$

The mean-field rate equations of epidemic spreading in layer B are thus given by

$$\frac{ds^B_{k_B}(t)}{dt} = -s^B_{k_B}(t)\beta_B k_B \Theta_B(t) - p\beta_A\Theta_A(t)\sum_{k_A} s^A_{k_A}(t) k_A P_A(k_A), \tag{S4}$$

$$\frac{d\rho^B_{k_B}(t)}{dt} = s^B_{k_B}(t)\beta_B k_B \Theta_B(t) - \rho^B_{k_B}(t), \tag{S5}$$

$$\frac{dr^B_{k_B}(t)}{dt} = \rho^B_{k_B}(t), \tag{S6}$$

$$\frac{dv^B_{k_B}(t)}{dt} = p\beta_A\Theta_A(t)\sum_{k_A} s^A_{k_A}(t) k_A P_A(k_A), \tag{S7}$$

where $\Theta_A(t)$ [$\Theta_B(t)$] is the probability that a neighboring node in layer A (layer B) is in the infected state.

From Eqs. (S1)-(S7), the density associated with each distinct state in layer A or B is given by

$$X_h(t) = \sum_{k_h=1}^{k_{h,max}} P_h(k_h) X^h_{k_h}(t). \tag{S8}$$

where $h \in \{A, B\}$, $X \in \{S, I, R, V\}$, and $k_{h,max}$ denotes the largest degree of layer h. The final densities of the whole system can be obtained by taking the limit $t \to \infty$.

B. Linear analysis for the information threshold

On an uncorrelated layered network, at the outset of the spreading dynamics, the whole system can be regarded as consisting of two coupled SI-epidemic subsystems [2] with the time evolution described by Eqs. (S2) and (S5). For $t \to 0$, we have $s^A_{k_A}(t) \approx 1$ and $s^B_{k_B}(t) \approx 1$, which reduce Eqs. (S2) and (S5) to

$$\begin{cases} \frac{d\rho^A_{k_A}(t)}{dt} = \beta_A k_A \Theta_A(t) + \beta_B \langle k_B \rangle \Theta_B(t) - \rho^A_{k_A}(t), \\ \frac{d\rho^B_{k_B}(t)}{dt} = \beta_B k_B \Theta_B(t) - \rho^B_{k_B}(t). \end{cases} \tag{S9}$$

For convenience, Eq. (S9) can be written concisely as

$$\frac{d\vec{\rho}}{dt} = C\vec{\rho} - \vec{\rho}, \tag{S10}$$

where the vector of infected density is defined as

$$\vec{\rho} \equiv (\rho^A_{k_A=1}, \dots, \rho^A_{k_{A,max}}, \rho^B_{k_B=1}, \dots, \rho^B_{k_{B,max}})^T,$$

and C is a block matrix in the following form:

$$C = \begin{pmatrix} C^A & D^B \\ 0 & C^B \end{pmatrix}, \tag{S11}$$

with matrix elements given by

$$\begin{aligned} C^A_{k_A,k'_A} &= [\beta_A k_A (k'_A - 1) P_A(k'_A)]/\langle k_A \rangle, \\ C^B_{k_B,k'_B} &= [\beta_B k_B (k'_B - 1) P_B(k'_B)]/\langle k_B \rangle, \\ D^B_{k_B,k'_B} &= \beta_B (k'_B - 1) P_B(k'_B). \end{aligned}$$

In general, information spreading on layer A can be facilitated by the outbreak of the epidemic on layer B, as an infected node in layer B instantaneously makes its counterpart node in layer A "infected" with the information about the disease. This coupling effect, in combination with the intrinsic spreading dynamics on layer A, leads to more informed nodes in the communication layer than infected nodes on layer B. If the maximum eigenvalue Λ_C of matrix C is greater than 1, an outbreak of the information will occur in the system [3]. We then have

$$\Lambda_C = \max\{\Lambda_A, \Lambda_B\}, \tag{S12}$$

where $\max\{\}$ denotes the greater of the two, and

$$\begin{aligned} \Lambda_A &= \beta_A(\langle k_A^2 \rangle - \langle k_A \rangle)/\langle k_A \rangle, \\ \Lambda_B &= \beta_B(\langle k_B^2 \rangle - \langle k_B \rangle)/\langle k_B \rangle \end{aligned}$$

are the maximum eigenvalues of matrices C^A and C^B [4], respectively. The outbreak threshold of information spreading in layer A is given by

$$\beta_{Ac} = \begin{cases} \beta_{Au}, & for \quad \beta_B \le \beta_{Bu} \\ 0, & for \quad \beta_B > \beta_{Bu} \end{cases} \tag{S13}$$

where $\beta_{Au} \equiv \langle k_A \rangle / (\langle k_A^2 \rangle - \langle k_A \rangle$ and $\beta_{Bu} \equiv \langle k_B \rangle / (\langle k_B^2 \rangle - \langle k_B \rangle$ denote the outbreak threshold of information spreading on layer A when it is isolated from layer B, and that of epidemic spreading on layer B when the coupling between the two layers is absent, respectively.

C. Competing percolation theory for epidemic threshold

To elucidate the interplay between epidemic and vaccination spreading, we must first determine which one is the faster "disease." At the early time of the epidemic outbreak on the isolated layer B, the average number of infected nodes grows exponentially as

$$N_e = n_0 R_e^t = n_0 e^{t \ln R_e}, \tag{S14}$$

where $R_e = \beta_B/\beta_{Bu}$ is the basic reproductive number for the disease on the isolated layer B [5], and n_0 denotes the number of initially infected nodes. Similarly, for information spreading on the isolated layer A, the average number of informed nodes at the early time is

$$N_i = n_0 R_i^t = n_0 e^{t \ln R_i}, \tag{S15}$$

where $R_i = \beta_A/\beta_{Au}$ is the reproductive number for information spreading on the isolated layer A. The resulting number of vaccinated nodes on layer B is

$$N_v = pn_0 R_i^t = pn_0 e^{t \ln R_i}. \tag{S16}$$

Since both epidemic and vaccination spreading processes exhibit exponential growth, we can obtain the ratio of their growth rates as

$$\theta = \frac{R_e}{R_i} = \frac{\beta_B \beta_{Au}}{\beta_A \beta_{Bu}}. \tag{S17}$$

For $\theta > 1$, i.e., $\beta_B \beta_{Au} > \beta_A \beta_{Bu}$, the epidemic disease spreads faster than the vaccination. In this case, the vaccination spread is insignificant and can be neglected.

To uncover the impact of information spreading on epidemic outbreak, we focus on the case of faster vaccination, i.e., $\theta < 1$, in accordance with the fact that information always tends to spread much faster than epidemic in a modern society. Given that vaccination and epidemic can be treated successively and separately, the threshold of epidemic outbreak can be derived by a bond percolation analysis [6, 7].

Firstly, when information spreading on layer A is over, the density of informed population is given by [5]

$$S_A = 1 - G_{A0}(u), \tag{S18}$$

where $G_{A0}(x) = \sum_{k_A} P_A(k_A) x^{k_A}$ is the generating function for the degree distribution of layer A, and u is the probability that a node is not connected to the giant cluster via a particular one of its edges, which can be solved by

$$u = 1 - \beta_A + \beta_A G_{A1}(u), \tag{S19}$$

where $G_{A1}(x) = \sum_{k_A} Q_A(k_A) x^{k_A}$ is the generating function for the excess degree distribution, $Q_A(k_A) = (k_A + 1) P_A(k_A + 1)/\langle k_A \rangle$, of layer A. Since p is the probability that an informed node in layer A makes its counterpart node in layer B vaccinated, the number of vaccinated or removed nodes in layer B is pS_A. A necessary condition for the outbreak of epidemic is the existence of a giant residual cluster in layer B [8]. We have

$$1 - pS_A > f_{Bc} = \frac{1}{G'_{B1}(1)}, \tag{S20}$$

where $G_{B1}(x) = \sum_{k_B} Q_B(k_B) x^{k_B}$ is the generating function for the excess degree distribution, $Q_B(k_B) = (k_B + 1) P_B(k_B + 1)/\langle k_B \rangle$, of layer B, and the prime denotes derivative. From Eq. (S20), we see that epidemic outbreak can occur only if $pS_A < 1 - 1/G'_{B1}(1)$.

The degree distribution of the residual network of layer B is given by [9, 10]

$$\widetilde{P}_B(\widetilde{k}_B) = f \sum_{k'_B = \widetilde{k}_B}^{\infty} P_B(k'_B) \binom{k'_B}{\widetilde{k}_B} (1 - f)^{k'_B - \widetilde{k}_B} f^{\widetilde{k}_B}, \tag{S21}$$

where $f = 1 - pS_A$ is the probability that a node is in the residual network. The generating function for the degree distribution of the residual network is then [6]

$$H_{B0}(x) = fG_{B0}(1 - f + fx), \tag{S22}$$

where $G_{B0}(x) = \sum_{k_B} P_B(k_B)x^{k_B}$ is the generating function for the degree distribution of layer B. The generating function for its excess degree distribution is

$$H_{B1}(x) = \frac{H'_{B0}(x)}{H'_{B0}(1)}. \tag{S23}$$

The basic reproductive number for a disease spreading over the residual network of layer B is then given by [5]

$$\widetilde{R}_i = \beta_B H'_{B1}(1). \tag{S24}$$

The epidemic threshold corresponds to the point $\widetilde{R}_i = 1$, and thus we have $\beta_{Bc} = 1/H'_{B1}(1)$. From Eqs. (S22)-(S24), we obtain the epidemic threshold β_{Bc} as

$$\beta_{Bc} = \frac{\langle k_B \rangle}{(1 - pS_A)(\langle k_B^2 \rangle - \langle k_B \rangle)}, \tag{S25}$$

where S_A is the density of the informed population, which can be obtained by solving Eqs. (S18) and (S19).

S2. Spreading dynamics on correlated double-layer networks

We assume that layer A has the same degree distribution as layer B. After a certain fraction q of pairs of nodes, one from each layer, have been randomly rematched, the conditional probability $P(k_B|k_A)$ can be written as

$$P(k_B|k_A) = qP_B(k_B) + (1-q)\delta_{k_B,k_A}, \tag{S26}$$

or

$$P(k_A|k_B) = qP_A(k_A) + (1-q)\delta_{k_A,k_B}. \tag{S27}$$

A. Mean-field rate equations

Using Eqs. (S1)-(S3), we can write the mean-field rate equations for information spreading on layer A as

$$\frac{ds^A_{k_A}(t)}{dt} = -s^A_{k_A}(t)\{\beta_A k_A \Theta_A(t) + \beta_B \Theta_B(t)\sum_{k_B} k_B[qP_B(k_B) + (1-q)\delta_{k_B,k_A}]\}, \tag{S28}$$

$$\frac{d\rho^A_{k_A}(t)}{dt} = s^A_{k_A}(t)\{\beta_A k_A \Theta_A(t) + \beta_B \Theta_B(t)\sum_{k_B} k_B[qP_B(k_B) + (1-q)\delta_{k_B,k_A}]\} - \rho^A_{k_A}(t), \tag{S29}$$

$$\frac{dr^A_{k_A}(t)}{dt} = \rho^A_{k_A}(t). \tag{S30}$$

Similarly, the mean-field rate equations for epidemic spreading on layer B are

$$\frac{ds^B_{k_B}(t)}{dt} = -s^B_{k_B}(t)\beta_B k_B \Theta_B(t) - p\beta_A \Theta_A(t)\sum_{k_A} s^A_{k_A}(t) k_A[qP_A(k_A) + (1-q)\delta_{k_A,k_B}], \tag{S31}$$

$$\frac{d\rho^B_{k_B}(t)}{dt} = s^B_{k_B}(t)\beta_B k_B \Theta_B(t) - \rho^B_{k_B}(t), \tag{S32}$$

$$\frac{dr^B_{k_B}(t)}{dt} = \rho^B_{k_B}(t), \tag{S33}$$

$$\frac{dv^B_{k_B}(t)}{dt} = p\beta_A \Theta_A(t)\sum_{k_A} s^A_{k_A}(t) k_A[qP_A(k_A) + (1-q)\delta_{k_A,k_B}]. \tag{S34}$$

Substituting Eqs. (S28)-(S34) into Eq. (S8), we can get the density associated with each distinct state in layer A or B.

B. Linear analysis for the information threshold

At the outset of the spreading dynamics, the whole system can be regarded as two coupled SI-epidemic subsystems [2] with the time evolution described by Eqs. (S29) and (S32). In the limit $t \to 0$, we have $s^A_{k_A}(t) \approx 1$ and $s^B_{k_B}(t) \approx 1$. Equations (S29) and (S32) can then be reduced to

$$\begin{cases} \frac{d\rho^A_{k_A}(t)}{dt} = \beta_A k_A \Theta_A(t) + \beta_B[q\langle k_B\rangle + (1-q)k_A]\Theta_B(t) - \rho^A_{k_A}(t), \\ \frac{d\rho^B_{k_B}(t)}{dt} = \beta_B k_B \Theta_B(t) - \rho^B_{k_B}(t). \end{cases} \tag{S35}$$

which can be written concisely as

$$\frac{d\vec{\rho}}{dt} = C\vec{\rho} - \vec{\rho}, \tag{S36}$$

where the matrix C has the same form as in Eq. (S11) and

$$\begin{aligned} C^{A}_{k_A,k'_A} &= [\beta_A k_A (k'_A - 1) P_A(k'_A)]/\langle k_A \rangle, \\ C^{B}_{k_B,k'_B} &= [\beta_B k_B (k'_B - 1) P_B(k'_B)]/\langle k_B \rangle, \\ D^{B}_{k_B,k'_B} &= \beta_B [q\langle k_B \rangle + (1-q) k_A](k'_B - 1) P_B(k'_B)/\langle k_B \rangle. \end{aligned}$$

The threshold of information outbreak is given by

$$\beta_{Ac} = \begin{cases} \beta_{Au}, & for \quad \beta_B \le \beta_{Bu}, \\ 0, & for \quad \beta_B > \beta_{Bu}, \end{cases} \tag{S37}$$

which is the same as Eq. (9) in the main text. As described in uncorrelated networks, there are two distinct mechanisms that can lead to the outbreak of information on layer A, and these hold for correlated layered-networks as well. For $\beta_B \le \beta_{Bu}$, only a small number of nodes in layer B are infected, so the impact of the disease on information-outbreak threshold on layer A is negligible. For $\beta_B > \beta_{Bu}$, epidemic spreading can result in the outbreak of information. In this case, the information-outbreak threshold is zero.

C. Competing percolation theory for epidemic threshold

For $\beta_A \le \beta_{Au}$, information itself cannot spread through the population. There is thus hardly any effect of the information layer on the epidemic spreading on layer B, and we have $\beta_{Bc} \approx \beta_{Bu}$. But for $\beta_A > \beta_{Au}$, the effect of information spreading on the epidemic threshold cannot be ignored. To assess quantitatively the influence, we focus on the case of faster information spread, i.e., $\beta_A \beta_{Bu} > \beta_B \beta_{Au}$, rendering applicable a bond percolation analysis similar to uncorrelated networks. Specifically, after information spreads on layer A, the percentage of nodes that get the information is S_A, and the density of recovered nodes of degree k_A is $r^A_{k_A} = 1 - u^{k_A}$, where u is the probability that a node is not connected to the giant cluster by a particular edge [Eq. (S19)]. Vaccinating a number of counterpart nodes results in the random removal of some edges which connect the vaccinated nodes with the remaining nodes [9, 10]. The probability $\widetilde{h}$ of an edge linking to a vaccinated node is

$$\widetilde{h} = \frac{p \sum_{k_B} [(1-q) r_{k_A} + q S_A] k_B P_B(k_B)}{\langle k_B \rangle}. \tag{S38}$$

The new degree distribution of the residual network on layer B is thus given by

$$\widetilde{P}_B(\widetilde{k}_B) = \sum_{k'_B = \widetilde{k}_B}^{\infty} \{1 - p[(1-q) r_{k_A} + q S_A]\} P_B(k'_B) \binom{k'_B}{\widetilde{k}_B} (1 - \widetilde{h})^{\widetilde{k}_B} \widetilde{h}^{k'_B - \widetilde{k}_B}. \tag{S39}$$

The requirement that a giant residual cluster exists is

$$\frac{\langle \widetilde{k}^2_B \rangle}{\langle \widetilde{k}_B \rangle} > 2, \tag{S40}$$

where $\langle \widetilde{k}_B \rangle$ and $\langle \widetilde{k}^2_B \rangle$ are the first and second moments of the degree distribution, respectively. Finally, we obtain the epidemic threshold as

$$\beta_{Bc} = \frac{\langle \widetilde{k}_B \rangle}{\langle \widetilde{k}^2_B \rangle - \langle \widetilde{k}_B \rangle}. \tag{S41}$$

S3. Simulation results

We first describe the simulation process of the two spreading dynamics on double-layer networks, and then demonstrate the validity of the theoretical analysis on uncorrelated networks with different network sizes and degree exponents, finally, we present results for SF-SF correlated networks.

A. Simulation process

To initiate an epidemic spreading process, a node in layer B is randomly infected and its counterpart node in layer A is thus in the informed state, too. The updating process is performed with parallel dynamics, which is widely used in statistical physics [11]. At each time step, we first calculate the informed (infected) probability $\pi_A = 1-(1-\beta_A)^{n_I^A}$ [$\pi_B = 1-(1-\beta_B)^{n_I^B}$] that each susceptible node in layer A (B) may be informed (infected) by its informed (infected) neighbors, where n_I^A (n_I^B) is the number of its informed (infected) neighboring nodes.

According to the dynamic mechanism, once node A_i is in the susceptible state, its counterpart node B_i will be also in the susceptible state. Considering the asymmetric coupling between the two layers in this case, both the information-transmission and disease-transmission events can hardly occur at the same time. Thus, with probability $\pi_A/(\pi_A + \pi_B)$, node A_i have a probability π_A to get the information from its informed neighbors in layer A. If node A_i is informed, its counterpart node B_i will turn into the vaccination state with probability p. With probability $\pi_B/(\pi_A + \pi_B)$, node B_i have a probability π_B to get the infection from its infected neighbors in layer B, and then node A_i also get the information about the disease.

In the other case that node B_i and its corresponding node A_i are in the susceptible state and the informed (or refractory) state respectively, only the disease-transmission event can occur at the time step. Thus, node B_i will be infected with probability π_B.

After renewing the states of susceptible nodes, each informed (infected) node can enter the recovering phase with probability $\mu_A = 1.0$ ($\mu_B = 1.0$). The spreading dynamics terminates when all informed (or infected) nodes in both layers are recovered, and the final densities R_A, R_B, and V_B are then recorded. The simulations are implemented using 30 different two-layer network realizations. The network size of $N_A = N_B = 2 \times 10^4$ and average degrees $\langle k_A \rangle = \langle k_B \rangle = 8$ are used for all subsequent numerical results, unless otherwise specified.

B. Uncorrelated double-layer networks

The effect of network size N on the information and epidemic outbreak thresholds is first studied. According to Eq. (S13), the behavior of the information threshold can be classified into two classes. For $\beta_B \leq \beta_{Bu}$, the disease transmission on layer B has little impact on the information threshold, as we have $\beta_{Ac} \approx \beta_{Au} = \langle k_A \rangle / (\langle k_A^2 \rangle - \langle k_A \rangle)$; while $\beta_{Ac} = 0.0$ for $\beta_B > \beta_{Bu}$. We here focus on the information threshold for $\beta_B \leq \beta_{Bu}$. From Figs. S1(a) and (c), we see that the theoretical predictions are basically accordant with the simulated thresholds for different network sizes. With the growth of network size, the information threshold decreases as $\langle k^2 \rangle$ of layer A increases [12]. According to Eq. (S25), the theoretical epidemic threshold can be predicted. For SF-ER double-layer networks, Figs. S1(b) and (d) shows that the simulated epidemic thresholds deviate slightly from the theoretical predictions. However, the larger deviations occur for the larger values of the vaccination rate p, e.g., $p = 0.9$ in Fig. S2, because the basic assumption of competing percolation theory is not strictly correct for the finite-size networks. As pointed out by Karrer and Newman [7], in the limit of large network size N, the vaccination and epidemic processes can be treated successively and separately. On the double-layer networks with finite network size, the effect of information spreading is somewhat over-emphasized. From Figs. S1 and S2, we also see that the discrepancy between the simulated and theoretical thresholds decreases with network size N.

We then investigate how the degree heterogeneity of layer A influences the information and epidemic outbreak thresholds by adjusting the exponent γ_A. The information thresholds for the different exponents of layer A are compared in Fig. S3(a), and the stronger heterogeneity of layer A (i.e., smaller γ_A) can more easily make the information outbreak. Fig. S3(b) shows that increasing the heterogeneity of layer A can slightly raise the epidemic

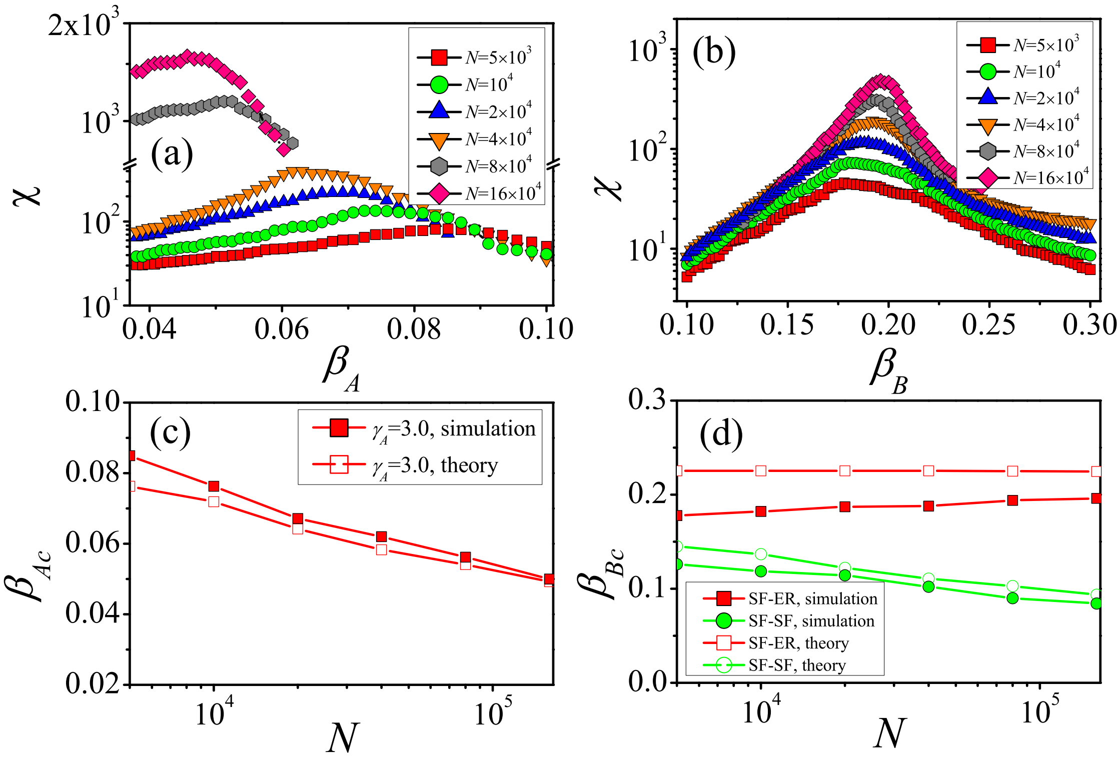

FIG. S 1. (Color online) On SF-ER networks, the susceptibility measure χ as a function of the information-transmission rate β_A at $\beta_B = 0.1$ (a) and the disease-transmission rate β_B at $\beta_A = 0.3$ (b) for $N = 5 \times 10^3$ (red squares), $N = 10^4$ (green circles), $N = 2 \times 10^4$ (blue up triangles), $N = 4 \times 10^4$ (orange down triangles), $N = 8 \times 10^4$ (gray hexagons) and $N = 16 \times 10^4$ (pink diamonds); (c) the information threshold β_{Ac} as a function of network size N at $\beta_B = 0.1$. (d) The epidemic threshold β_{Bc} as a function of N at $\beta_A = 0.3$ for SF-ER networks (red solid squares) and SF-SF networks (green solid circles). The same hollow symbols represent the corresponding theoretical thresholds. The other parameters are the degree exponent $\gamma_A = 3.0$ (or $\gamma_B = 3.0$) and vaccination rate $p = 0.5$.

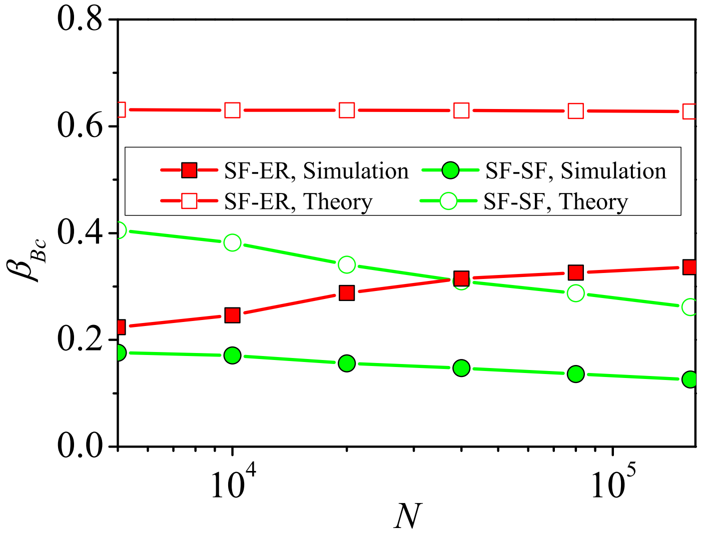

FIG. S 2. (Color online) The epidemic threshold β_{Bc} as a function of network size N for SF-ER networks (red solid squares) and SF-SF networks (green solid circles) at $\beta_A = 0.3$ and $p = 0.9$. The same hollow symbols represent the corresponding theoretical thresholds. For each SF layer, the degree exponent is set to $\gamma_A = 3.0$ (or $\gamma_B = 3.0$).

threshold β_{Bc} at a small information-transmission rate β_A, while making for the epidemic outbreak at a large β_A. This phenomenon results from the different effects of the heterogeneity on the information spreading under different transmission rates. The more homogeneous degree distribution does not always hinder the diffusion of information, especially at a large transmission rate [10, 13].

To further demonstrate the validity of the theoretical analysis, we consider the case of SF-SF double-layer networks. Similar to the case of SF-ER networks, the gap between the theoretical and simulated thresholds is narrowing with the increase of network size [see Figs. S1(d) and S2], which implies the reasonability of the assumption in the thermodynamic limit. The final dynamical state of the SF-SF spreading system is also shown in Fig. S4, and it displays a similar phenomenon to the case of SF-ER networks. We also see that the theoretical predictions from mean-field rate equations are in good agreement with the simulation results.

C. Correlated double-layer networks

On SF-SF correlated networks, we investigate the effect of positive inter-layer correlation on the two types of spreading dynamics. As shown in Figs. S5, S6 and S7, with the increase of the correlation m_s (by reducing the rematching probability q), the information threshold remains unchanged but the epidemic threshold can be enhanced, making the contact layer more robust to epidemic outbreak, which is consistent with the results for ER-ER correlated networks.

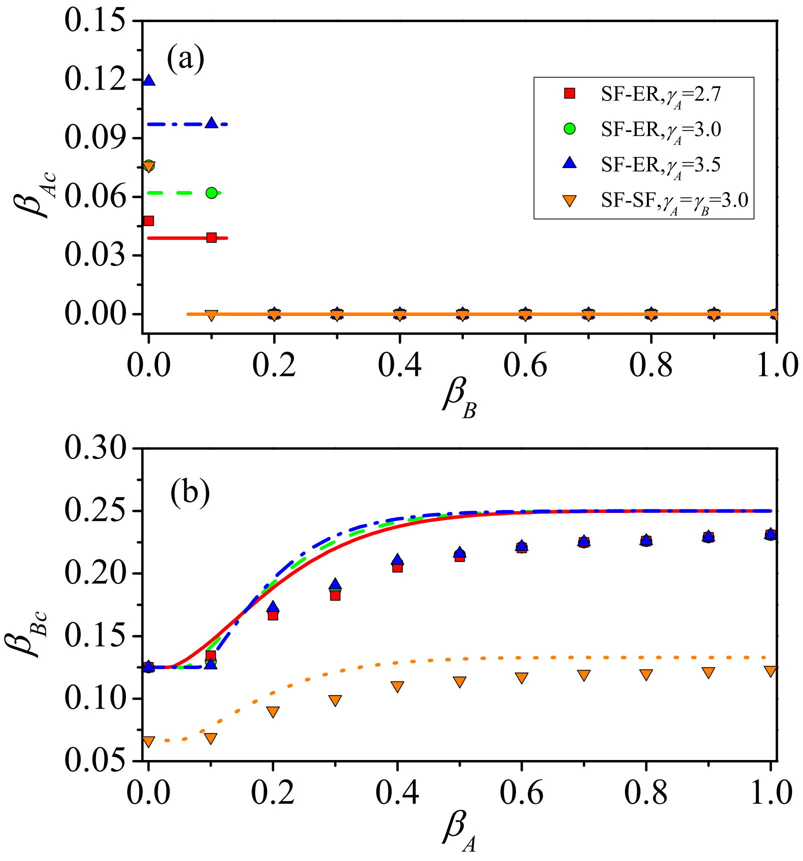

FIG. S 3. (Color online) On various double-layer networks, β_{Ac} versus β_B (a) and β_{Bc} versus β_A (b) for the SF-ER networks with $\gamma_A = 2.7$ (red squares), the SF-ER networks with $\gamma_A = 3.0$ (green circles), the SF-ER networks with $\gamma_A = 3.5$ (blue up triangles) and the SF-SF networks with $\gamma_A = \gamma_B = 3.0$ (orange down triangles). The analytical predictions of β_{Ac} and β_{Bc} are from Eq. (S13) and Eq. (S25), respectively. The vaccination rate is set to $p = 0.5$.

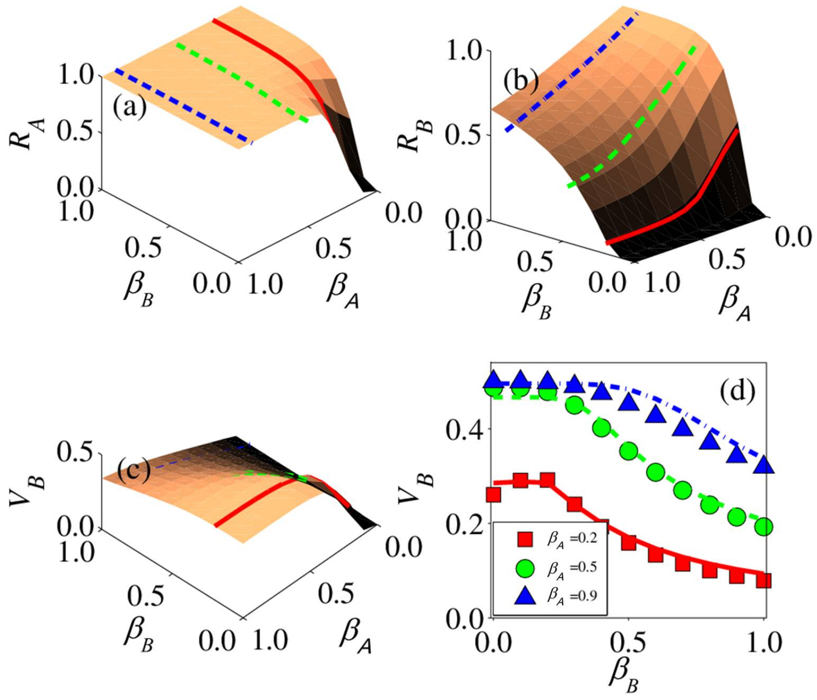

FIG. S 4. (Color online) For SF-SF networks, the final density in each state versus the parameters β_A and β_B: (a) recovered density R_A, (b) recovered density R_B, (c) the vaccination density V_B, and (d) V_B versus β_B for $\beta_A = 0.2, 0.5, 0.9$. The other parameter are $p = 0.5$ and $\gamma_A = \gamma_B = 3.0$. Different lines are the numerical solutions of Eqs. (S1)-(S8) in the limit $t \to \infty$. In (a) and (d), we select three different values of β_A (0.2, 0.5, and 0.9), corresponding to the red solid, green dashed, and blue dot-dashed lines, respectively. In (b) and (c), three different values of β_B are chosen (0.2, 0.5, and 0.9), corresponding to the red solid, green dashed, and blue dot-dashed lines, respectively.

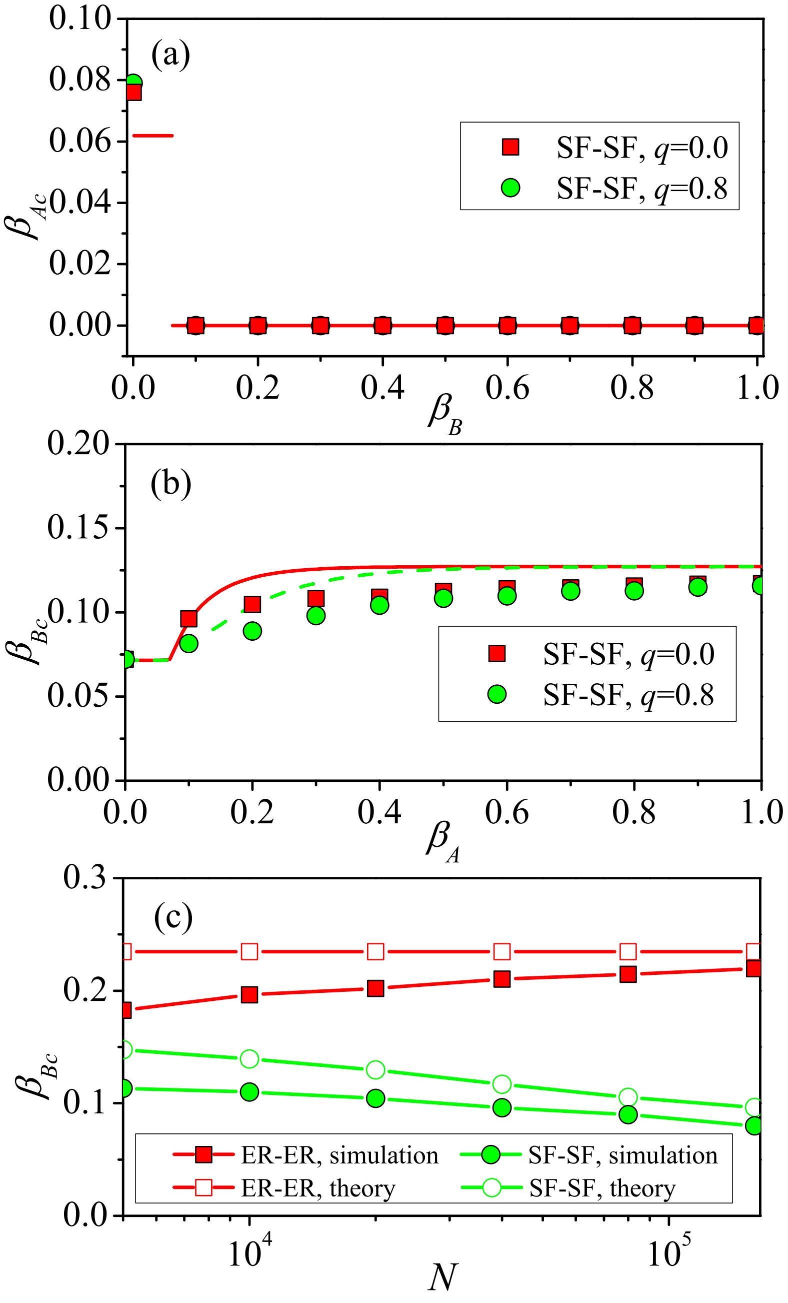

FIG. S 5. (Color online) On double-layer networks, (a) β_{Ac} versus β_B, (b) β_{Bc} versus β_A, and (c) β_{Bc} versus N at $\beta_A = 0.3$. In (a) and (b), red solid squares and green solid circles respectively denote the simulation results for $q = 0.0$ and $q = 0.8$ on SF-SF networks, and the lines are the corresponding theoretical thresholds. In (c), the value of parameter q is 0.0, solid red squares and solid green circles respectively represent the results for ER-ER and SF-SF networks, and the same shapes are the corresponding theoretical predictions. The other parameter are $p = 0.5$ and $\gamma_A = \gamma_B = 3.0$.

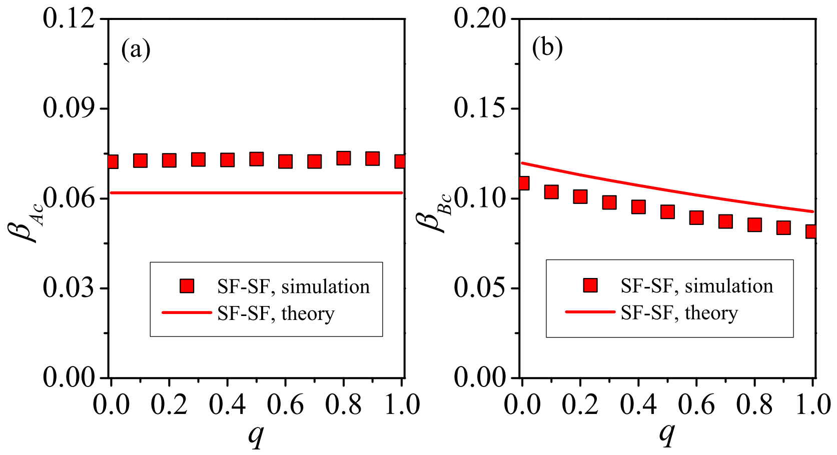

FIG. S 6. (Color online) On SF-SF networks, the effect of varying the rematching probability on outbreak thresholds of the two types of spreading dynamics. (a) β_{Ac} versus q for $\beta_B = 0.05$ and $p = 0.5$. Red Solid line is the analytical prediction from Eq. (S37). (b) β_{Bc} versus q for $\beta_A = 0.2$ and $p = 0.5$. Red solid line is the analytical prediction from Eqs. (S38)-(S41). The value of degree exponent is $\gamma_A = \gamma_B = 3.0$.

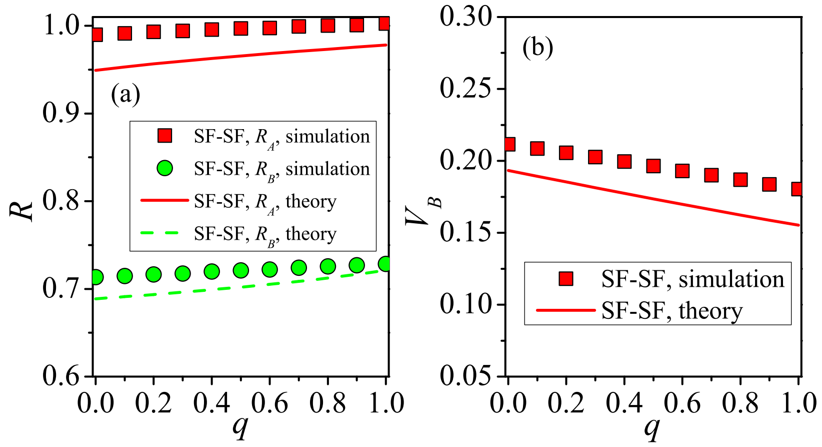

FIG. S 7. (Color online) On SF-SF networks, the effect of rematching probability on the final state. (a) R_A versus q (red squares) and R_B versus q (green circles), (b) V_B versus q (red squares). Different lines represent the analytic solutions for SF-SF networks, calculated by summing the final densities of all degrees from Eqs. (S28)-(S34). The parameter setting is $\gamma_A = \gamma_B = 3.0, \beta_A = 0.2$, $\beta_B = 0.4$ and $p = 0.5$.

References

[1] Barthélemy, M., Barrat, A., Pastor-Satorras, R. & Vespignani, A. Velocity and Hierarchical Spread of Epidemic Outbreaks in Scale-Free Networks. *Phys. Rev. Lett.* **92**, 178701 (2004).

[2] Newman, M. E. J. *Networks An Introduction* (Oxford University Press, Oxford, 2010).

[3] Saumell-Mendiola, A., Ángeles Serrano, M. & Boguñá, M. Epidemic spreading on interconnected networks. *Phys. Rev. E* **86**, 026106 (2012).

[4] Mieghem, P. V. *Graph Spectra for Complex Networks* (Cambrige university press, England, 2011).

[5] Newman, M. E. J. Spread of epidemic disease on networks. *Phys. Rev. E* **66**, 016128 (2002).

[6] Newman, M. E. J. Threshold Effects for Two Pathogens Spreading on a Network. *Phys. Rev. Lett.* **95**, 108701 (2005).

[7] Karrer, B. & Newman, M. E. J. Competing epidemics on complex networks.*Phys. Rev. E* **84**, 036106 (2011).

[8] Gao, J., Buldyrev, S. V., Stanley, H. E. & Havlin,S. Networks formed from interdependent networks. *Nat. Phys.* **8**, 40-48 (2012).

[9] Cohen, R., Erez, K., ben-Avraham, D., & Havlin, S. Breakdown of the Internet under Intentional Attack. *Phys. Rev. Lett.* **86**, 3682 (2001).

[10] Pastor-Satorras, R. & Vespignani, A. Immunization of complex networks. *Phys. Rev. E* **65**, 036104 (2002).

[11] Marro, J. & Dickman, R. *Nonequilibrium Phase Transitions in Lattice Models* (Cambridge University Press, Cambridge, 1999).

[12] Boguñá, M., Castellano, C. & Pastor-Satorras, R. Nature of the Epidemic Threshold for the Susceptible-Infected-Susceptible Dynamics in Networks. *Phys. Rev. Lett.* **111**, 068701 (2013).

[13] Pastor-Satorras, R., & Vespignani, A. Epidemic dynamics and endemic states in complex networks. *Phys. Rev. E* **63**, 066117 (2001).
